# Application of metabolite set enrichment analysis on untargeted metabolomics data prioritises relevant pathways and detects novel biomarkers for inherited metabolic disorders

**DOI:** 10.1002/jimd.12522

**Published:** 2022-05-22

**Authors:** Brechtje Hoegen, Juliet E. Hampstead, Udo F.H. Engelke, Purva Kulkarni, Ron A. Wevers, Han G. Brunner, Karlien L. M. Coene, Christian Gilissen

**Affiliations:** ^1^ Department of Human Genetics, Radboud Institute for Molecular Life Sciences Radboud University Medical Center Nijmegen The Netherlands; ^2^ Department of Laboratory Medicine, Translational Metabolic Laboratory (TML) Radboud University Medical Center Nijmegen The Netherlands; ^3^ Department of Clinical Genetics, Maastricht University Medical Center, GROW School of Oncology and Development, MHENS School of Neuroscience Maastricht University Maastricht The Netherlands

**Keywords:** biochemical pathways, biomarkers, cystathionine ß‐synthase, inborn errors of metabolism, inherited metabolic disorders, mass spectrometry, metabolite set enrichment analysis, next‐generation metabolic screening, untargeted metabolomics

## Abstract

Untargeted metabolomics (UM) allows for the simultaneous measurement of hundreds of metabolites in a single analytical run. The sheer amount of data generated in UM hampers its use in patient diagnostics because manual interpretation of all features is not feasible. Here, we describe the application of a pathway‐based metabolite set enrichment analysis method to prioritise relevant biological pathways in UM data. We validate our method on a set of 55 patients with a diagnosed inherited metabolic disorder (IMD) and show that it complements feature‐based prioritisation of biomarkers by placing the features in a biological context. In addition, we find that by taking enriched pathways shared across different IMDs, we can identify common drugs and compounds that could otherwise obscure genuine disease biomarkers in an enrichment method. Finally, we demonstrate the potential of this method to identify novel candidate biomarkers for known IMDs. Our results show the added value of pathway‐based interpretation of UM data in IMD diagnostics context.

## Graphical abstract

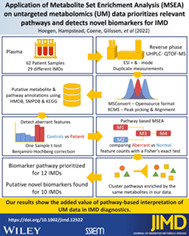

## 1 INTRODUCTION

Inherited metabolic disorders (IMDs) are typically diagnosed via a combination of genetic and biochemical tests. These approaches are generally very specific, testing the activity of a single enzyme or a preselected set of metabolites. The high heterogeneity, immediate postnatal presentation, and nonspecific symptomology of IMDs make the selection of an adequate panel of targeted metabolic tests challenging. Important diagnostic biomarkers can be absent from the panel if they were identified recently, and there is no opportunity for the detection of novel biomarkers.

Recent advances in metabolomic methods have enabled the use of untargeted metabolomics (UM) as a viable alternative to targeted approaches.^1^ We have coined UM in the context of diagnostic screening for IMDs as next‐generation metabolic screening (NGMS).^2^ NGMS allows the simultaneous measurement of hundreds of metabolites, circumventing the need for targeted metabolic tests based on patient phenotype as a first‐tier screening for IMDs. Several studies have demonstrated the effectiveness of NGMS for screening IMDs in individual patients with a well‐described set of diagnostic biomarkers.^2^, ^3^, ^4^, ^5^, ^6^, ^7^ However, many patients still lack a definitive diagnosis when restricting the analysis to known biomarkers. Looking for ways to systematically analyse a larger set of metabolites present within the NGMS data is the next logical step.

The biggest challenge in the identification of novel biomarkers in NGMS data is the prioritisation of relevant metabolites from the hundreds or thousands that are identified. Prioritisation based on peak intensity, fold change, and case–control statistics is useful, but disease relevant metabolites can sometimes be concealed by metabolite aberrations caused by confounding factors such as diet or medication.^8^ To improve prioritisation, biological network relationships can be leveraged. Defective enzymes in IMD patients are often members of biochemical pathways, with each enzyme in the pathway representing a catalytic step dependent on substrates from the preceding steps. The defective enzyme will perturb both downstream and upstream reactions in pathways. We hypothesise that these perturbations may be detectable in NGMS data and can be utilised to improve the diagnostics process in IMD patients and facilitate the identification of novel biomarkers.

To this end, we implemented a metabolite set enrichment analysis (MSEA) method,^1^, ^9^, ^10^ that enables the high‐throughput analysis of UM data. MSEA identifies small sets of pathway‐associated aberrant metabolites from the large number of aberrant features present in a sample using a statistical enrichment‐based approach.^1^, ^9^, ^10^ We show that incorporation of pathway context contributes to prioritisation of biomarkers in IMD patients. Furthermore, we show the potential of MSEA for the identification of putatively novel biomarkers and suggest several candidate biomarkers for known IMDs. Finally, we demonstrate that collective analysis of pathway enrichment across our data cohort can help to distinguish IMD‐specific from IMD‐unspecific pathway enrichment.

## 2 METHODS

### 2.1 UM Data

The data used in this study were described previously by Coene et al.^2^ Briefly, NGMS data were generated from plasma samples using reverse phase ultra‐high‐performance liquid chromatography coupled with electrospray ionisation quadrupole time‐of‐flight mass spectrometry (QTOF‐MS). Not all data from the study^2^ were included for the current study; data were only included when controls were present in the batch and all measurements present in the batch passed quality control. More details on data exclusion can be found in Table S1A. Data from 62 samples, representing 55 patients, covering 29 IMDs, were included for the current study (Table 1). The patient data were retrospectively gathered from 15 batches of NGMS data measured between 2012 and 2017. The NGMS data generated before April 2016 were measured on the Agilent 6540 QTOF‐MS (11 batches) and later data were measured on the 6545 QTOF‐MS (4 batches). Although the Agilent 6545 QTOF‐MS generates a larger number of significant features, our previous study shows that the diagnostic outcome was unaffected by the different QTOF‐MS instruments.^2^ Each analytical batch contained approximately 10 control samples. All included samples were measured in duplicate. For some patient data, additional technical and biological replicates were available. Multiple samples were measured for four patients and seven samples were measured in multiple batches. An overview of included patient data can be found in Table S1B.

**TABLE 1:** Samples and biomarker counts by inherited metabolic disorder (IMD). Samples in our cohort (*n* = 62) are summarised by the diagnosed IMD (left), IMD OMIM ID. We also list the number of theoretical biomarkers per IMD used in our analysis, separated into all 102 known biomarkers (left) and 54 biomarkers associated with a Kyoto Encyclopedia of Genes and Genomes (KEGG) or Small Molecule Pathway Database (SMPDB) pathway (right). Grey lines indicate that biomarker pathways were enriched for all cohort samples of the IMD indicated with the exception of phenylketonuria, for which biomarker pathways were enriched in only 8 out of 9 samples

		Biomarkers		Samples
IMD	OMIM	Total	Pathway	
3‐Ureidopropionase deficiency	613161	4	4	1
3β‐Hydroxy‐∆5‐C27‐steroid dehydrogenase deficiency	607765	5	1	1
ACSF3 deficiency (CMAMMA)	614265	2	2	3
Adenylosuccinate lyase deficiency	103050	2	1	1
Alkaptonuria	203500	1	1	1
Aminoacylase I deficiency	609924	10	1	2
Cystathionine ß‐synthase deficiency	236200	2	2	1
Dimethylglycine dehydrogenase deficiency	605850	1	1	1
Glutamate formimino transferase deficiency	229100	2	2	1
Glutaric aciduria Type I	231670	2	1	1
Guanidinoacetate methyltransferase deficiency	601240	3	3	1
Histidinemia	235800	2	2	1
Hyperlysinemia, Type I	238700	3	2	2
Hyperprolinemia, Type II	239510	4	3	2
Maple syrup urine disease	248600	7	5	2
Methionine Adenosyltransferase I/III deficiency	250850	3	1	2*
Molybdenum cofactor deficiency	252150	5	5	2
NANS deficiency	610442	1	1	1
Ornithine aminotransferase deficiency	258870	3	2	1
Phenylketonuria	261600	5	3	9*
Pyridoxine‐dependent epilepsy	266100	3	3	1
UMP synthase deficiency	258900	2	2	1
Xanthinuria, Type II	603592	6	6	2
(Very‐)Long‐chain acyl‐CoA dehydrogenase deficiency	201475	3	0	1
3‐Hydroxy‐3‐methylglutaryl‐CoA lyase deficiency	246450	6	0	5*
3‐Ketothiolase deficiency	203750	4	0	1
3‐Methylcrotonyl‐CoA carboxylase deficiency	210200	3	0	5
Cerebrotendinous xanthomatosis	213700	1	0	5
Medium‐chain acyl‐CoA dehydrogenase deficiency	201450	7	0	5*
Total		102	54	62

* Some of these samples belong to the same patient, see Table S2 for more details.

### 2.2 Preprocessing

MSConvert was used to convert data to mzML format (ProteoWizard version 3.0.19161).^11^ Feature detection and alignment were performed by XCMS (R version 3.6.1 and xcms version 3.4.4).^12^ The parameter settings used for MSConvert and XCMS can be found in Table S2A,B.

### 2.3 Aberrant feature detection

An in‐house diagnostic pipeline as described by Coene et al.^2^ and Hoegen et al.^13^ was used for detecting aberrant features based on feature intensity. However, the Bonferroni–Holm correction method in the original pipeline was replaced with the less stringent Benjamini–Hochberg correction, so more features could be taken along for our enrichment analysis. Features were considered aberrant if they differed significantly (*α* < 0.05) between the patient and controls.

### 2.4 Metabolite and pathway annotations

Neutral masses were estimated by correcting the feature m/z for mH^+^, mNa^+^, mH^−^ and mCl^−^ adducts in their respective ion modes. Features were then assigned putative metabolite annotations by searching the Human Metabolome Database (HMDB; containing 114 003 metabolites as of June 19, 2018)^14^ and Kyoto Encyclopedia of Genes and Genomes (KEGG; containing 17 980 metabolites as of May 7, 2017)^15^ databases with the estimated neutral mass, tolerating at most 5 ppm difference. Often assigning multiple putative annotations per feature (mean = 2.31; SD = 2.39). Next, features were mapped to biological pathways using their assigned metabolite annotations, HMDB identifiers were coupled to Small Molecule Pathway Database (SMPDB; containing 894 primary pathways as of September 14, 2018)^16^ pathways and KEGG identifiers were coupled to the KEGG pathways (containing 317 human pathways as of May 7, 2017). HMDB annotations are specifically designed for the human metabolome and SMPDB contains a significant number of IMD pathways (Table S3A), while KEGG is a more generalised database encompassing a wide variety of biological compounds and pathways.

### 2.5 Metabolite set enrichment analysis

MSEA was built using Java version jre1.8.0_121. The code and README will be publicly available on GitHub: github.com/Genome-Bioinformatics-RadboudUMC/MetaboliteSetEnrichmentAnalysis. Statistical enrichment of pathways was computed using a one‐sided Fisher's exact test comparing aberrant pathway‐associated and pathway‐unassociated features with their non‐aberrant counterparts. Only pathways associated with >1 aberrant features were considered. Fisher's *p*‐values were Bonferroni–Holm corrected for multiple testing by the total number of tested pathways, and those that remained significant at *p* < .05 were retained.

### 2.6 Clustering of enriched pathways

Many of the pathways available in SMPDB and KEGG contain partially overlapping processes or share many metabolites. This overlap can cause redundant enriched pathways in MSEA output, for example, due to a single set of features enriching several pathways with a high degree of similarity to each other. To group pathways enriched by the same set of metabolites, a clustering approach was applied. Clustering was performed based on shared aberrant metabolites, and enriched pathways with 100% overlap (in either direction) were clustered together. Metabolite IDs between HMDB and KEGG are not well connected; therefore, pathways from either database were clustered separately. But pathway clusters with similar aberrant features are mentioned in the output file. This clustering step serves to group pathways with the same root metabolite set driving enrichment. For each cluster of pathways, we selected the most descriptive pathway through following steps: (1) select pathways that are associated to the highest number of aberrant features; (2) choose the pathway with lowest *p*‐value; (3) when available, give preference to pathways categorised as “Metabolic” in SMPDB and “Metabolism” in KEGG.

### 2.7 Metabolite biomarkers, biomarker pathways and biomarker clusters

For each of the 29 IMDs included in this study one or more metabolite biomarkers are known (Tables 1, S3B).^2^ When a pathway contains one or more metabolite biomarkers for a specific IMD, that pathway is considered to be a biomarker pathway for that IMD. For 23 IMDs such biomarker pathways are available (Table 1; pathway biomarkers >0, Table S3C). A cluster of pathways that contains biomarker pathways is considered to be a biomarker cluster.

### 2.8 Biomarker rank

To assess how well MSEA prioritises IMD biomarkers, we determined the position at which each biomarker ranks at feature, pathway and pathway cluster level. To determine the biomarker rank at feature level, the aberrant feature list was sorted based on feature intensity. To determine the pathway feature rank and cluster feature rank, features were first sorted by pathway or pathway cluster *p*‐value and then by feature intensity, with aberrant features in the most enriched pathways or pathway clusters prioritised. Across all three sets, the highest ranked feature associated with a biomarker metabolite was selected. Feature intensity was chosen for ranking as receiver operating characteristic curves showed that it was a better binary classifier of biomarker status in our data than fold change and *p*‐value (Figure S1). We describe these ranks as row indices, where a row index of 1 indicates the feature is ranked first of all features.

We additionally determined pathway biomarker ranks for pathways and pathway clusters. To do this, we sorted all enriched pathways and pathway clusters by their respective *p*‐value, and determined the ranks of pathways or pathway clusters containing biomarker metabolites. Similar to the previous method, the lowest ranked pathway or pathway cluster containing a biomarker metabolite was selected.

### 2.9 Manual analysis of the cystathionine ß‐synthase‐deficiency patient

The cystathionine ß‐synthase (CBS)‐deficiency patient was manually analysed to demonstrate the results given by MSEA in more detail. Incorrect metabolite annotations were determined for the CBS‐deficiency patient as following: (1) When the retention time (RT) for a metabolite was known on our LC system (via the use of model compounds or diagnosed patient samples; see Reference 2 for more details), we checked if the known metabolite RT matched with the feature's RT (RT difference **≤**0.1 minutes). (2) The feature's presence was checked in the raw data, to determine potential alignment errors. (3) The laboratory technician's expertise in biochemistry and clinical chemistry was used to assess the likelihood of the metabolite annotation.

## 3 RESULTS

Sixty‐two samples were included in this study, representing 55 patients and spanning 29 distinct IMDs. These samples were further divided across 15 analytical batches (4.6 mean samples per batch, 4.9 SD samples per batch), and 2 Agilent instrument types (6540 QTOF: 53 samples; 6545 QTOF: 15 samples, 6 of which were technical replicates run on both instruments). Sample counts per IMD are summarised in Table 1 alongside the number of known IMD biomarkers used in this study; a complete measurement list is provided in Table S1, and a list of IMD biomarkers and corresponding pathways are provided in Table S3 (B, biomarkers; C, biomarker pathways). A median of 29 394 features were present per sample of which a median of 616 were aberrant. A median of 299 aberrant features received 1 or more metabolite annotations and a median of 103 aberrant features were associated to one or more pathways through one or more metabolite annotations (Figure S2).

**FIGURE 1:**
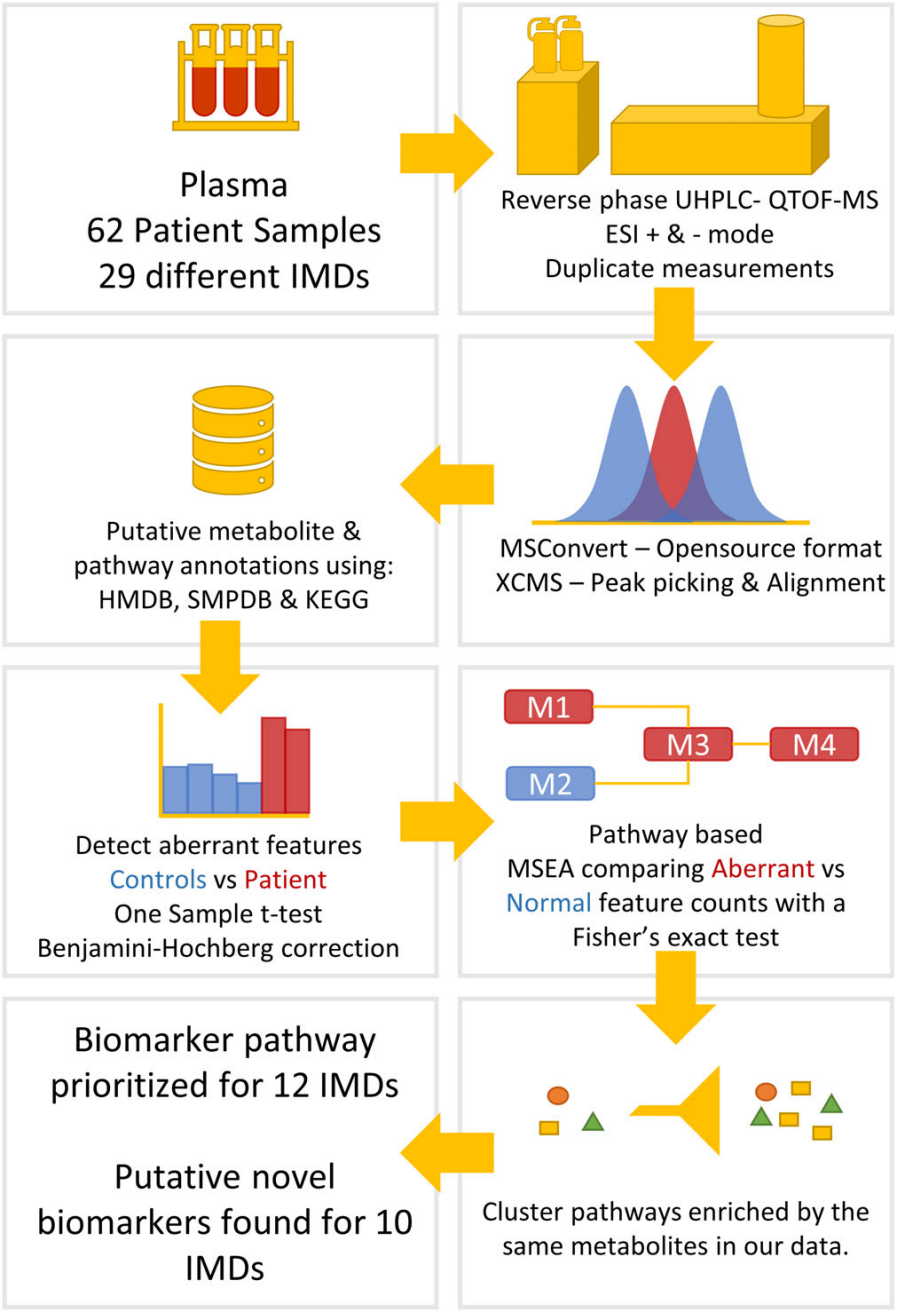
Metabolite set enrichment analysis (MSEA) method overview. Visualisation of our untargeted metabolomics workflow, including MSEA. Analysis up to aberrant feature detection was performed using an in‐house pipeline described previously by Coene et al.^2^ Adjustments made to the in‐house pipeline and the implementation of MSEA and clustering steps are described in more detail in the materials and methods

To calculate enrichment in biological pathways, we used the primary pathways from the Small Molecule Pathway Database (SMPDB, 894 pathways) and the human pathways from Kyoto Encyclopedia of Genes and Genomes (KEGG, 317 pathways). The method, process, and output of MSEA described here is summarised in Figure 1. From the 62 samples included, 19 samples (covering 6 IMDs) were excluded from further analysis on grounds that no biomarker pathways (see Section 2.7) were available in SMPDB or KEGG (Table 1; pathway biomarkers = 0). Another 5 samples had no significantly enriched pathways following MSEA. However, for 1 of these 5 samples a replicate measurement from the new more sensitive QTOF‐MS instrument (QTOF 6545) was available, for which pathways were found to be enriched by MSEA. From the remaining 39 samples, for which MSEA output and biomarker pathways were available, 26 (covering 13 IMDs) had at least one biomarker pathway enriched (Tables 3 and S3C). For Alkaptonuria, the metabolite aberrations in the biomarker pathway were caused by the patient's treatment.

**FIGURE 2:**
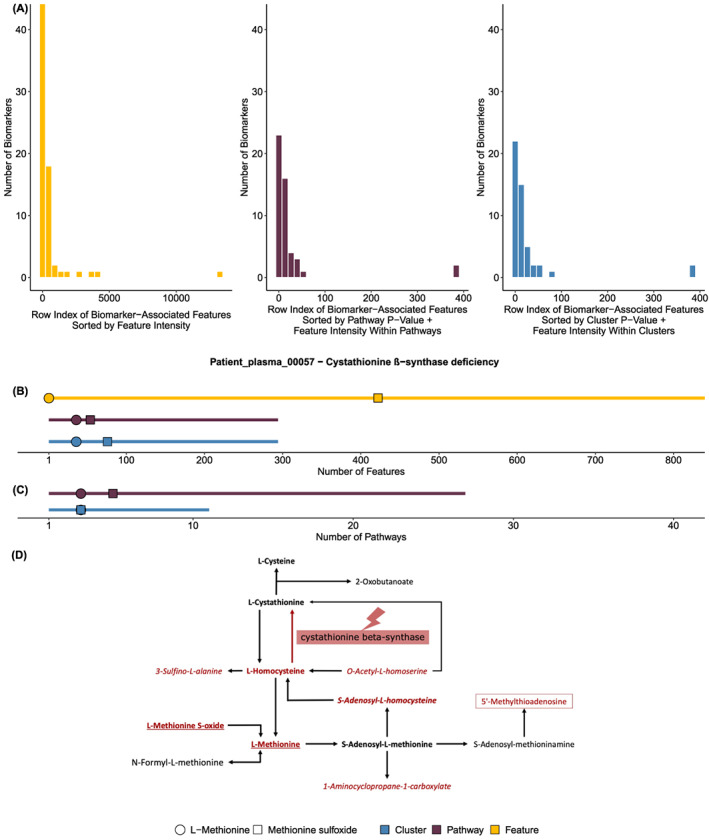
Metabolite set enrichment analysis (MSEA) prioritises known inherited metabolic disorder (IMD) biomarkers. (A) The row index distribution of all biomarker‐associated features are shown ranked by feature intensity (yellow), MSEA pathway *p*‐value (purple) and MSEA cluster *p*‐value (blue; see Section 2). (B) An example patient (00057) with cystathionine ß‐synthase (CBS) deficiency is shown. The number of biomarker‐associated features in each category (significant features, yellow; significant features within an MSEA‐enriched pathway, purple; significant features within an MSEA cluster, blue) is indicated by a line. Along this line, biomarker‐associated features are shown; their position indicates where they rank in the feature distribution sorted as described in (A). (C) The same example patient (00057) with CBS deficiency is shown. Here the length of the line represents the number of pathways (purple) or clustered pathways (blue) and points represent the distribution of known biomarkers in these pathways ranked by pathway *p*‐value (purple) or cluster *p*‐value (blue, see Section 2). (D) A pathway depicting what metabolites we found to be aberrant in a CBS deficiency patient, who were in close proximity to CBS. Bold metabolites are known biomarkers on IEMbase, underlined metabolites are part of our own metabolite panel, red metabolites have aberrant features associated to them in our data and metabolites in italics are false positive hits in the pathway as the associated aberrant feature was incorrectly annotated, see Table S4C–F for more details.

MSEA effectively prioritises relevant biomarkers by biomarker rank (see Section 2). Prioritisation by feature intensity gave a median biomarker rank of 44 out of a median 1589 aberrant feature‐metabolite pairs per sample, whereas prioritisation by pathway *p*‐value followed by feature intensity gave a median feature biomarker rank of 4 out of a median 519 aberrant pathway‐associated feature‐metabolite pairs per sample (Table S3D). However, 23 biomarkers were not associated with enriched pathways and these were not prioritised by MSEA. To correct for this potential bias, we also considered only the median biomarker rank of pathway‐associated biomarkers by feature intensity (median = 14). All three approaches prioritised biomarker‐associated features significantly better than would be expected by chance (permutation test, *p* = 3.4E‐9, *p* = 1.56E‐6, *p* = 4.54E‐4). The distributions of biomarker ranks are shown in Figure 2A. Using these feature‐based ranks, we also show that MSEA prioritised pathway‐associated IMD biomarkers better than solely filtering by feature intensity (Wilcoxon *p* = 0.032; Figure S3). Clustering to eliminate pathways enriched by the same aberrant features did not reduce biomarker rank (median = 6, Wilcoxon *p* = 0.13, Figure S3). However, it did reduce the number of pathways so biomarker‐containing pathways were better prioritised (median = 21 pathways across all samples, median = 6 clusters across all samples; Wilcoxon *p* = 0.00075, Figure S4). Amongst enriched pathways and pathway clusters, the median biomarker rank was 1 (Figure S5).

To demonstrate how MSEA affects biomarker prioritisation, we have taken a closer look at the CBS‐deficiency patient in our cohort. In total 609 features were found to be aberrant, 337 of which could be assigned putative metabolite annotations and 60 could be assigned pathway annotations (see Section 2). For CBS deficiency, the NGMS data were screened for two biomarkers, l‐methionine (KEGG, C00073; HMDB, HMDB0000696) and l‐methionine sulphoxide (KEGG, C02989; HMDB, HMDB0002005), both were present in pathways (Tables 1 and S3B). Two features were found to be associated with l‐methionine, ranking on the first (ESIpos_981) and third (ESIneg_743) positions of the feature list (Figure 2B). For l‐methionine sulphoxide, we found one associated feature ranking on the 28th (ESIpos_1299) position of the feature list. Pathway feature rank and cluster feature rank (see Section 2) were slightly improved from the original feature rank (Figure 2B). After MSEA, 27 pathways were found to be enriched (Table S4A). The highest ranking biomarker pathway was located on Position 3, containing l‐methionine (Figure 2C). The first pathway containing both biomarkers was found on Position 5. In total, 12 enriched pathways contained one or both of our biomarkers, indicating that many of the enriched pathways may represent redundant processes. When clustering the pathways together that were enriched by the exact same set of aberrant metabolites, we were left with 11 clusters of pathways (Table S4B), 3 of which are biomarker clusters (see Section 2, Figure 2C). The highest ranking biomarker cluster was located on Position 3 of the pathway cluster list and contained all biomarkers. The two pathways ranking higher than biomarker pathways are hypothesised to be unrelated to CBS deficiency, but further work on samples from other CBS patients is required to confirm this with certainty (Table S4A).

Looking at aberrant metabolites for the CBS‐deficiency sample in the cysteine and methionine metabolism pathway (KEGG ID: hsa00270) (Figure 2D), we found metabolites aberrant that were not present in our biomarker panel (Table S4C). Several of the aberrant metabolites, homocysteine and S‐adenosylhomocysteine, are already known as biomarkers for CBS deficiency in IEMbase.^17^ However, S‐adenosylhomocysteine turned out to be an incorrect metabolite annotation (Table S4C). Four metabolites were marked to be aberrant that are currently not known to be biomarkers (Figure 2C). Manual checks in the raw data showed that the features in our data associated with 1‐aminocyclopropane‐1‐carboxylate (KEGG ID: C01234), *O*‐acetyl‐l‐homoserine (KEGG ID: C01077) and 3‐sulfinoalanine (KEGG ID: C00606), were incorrect results as they were either misannotated or misaligned (Table S4D–F). The feature associated with 5′‐methylthioadenosine (KEGG ID: C00170) is likely a putative novel biomarker for CBS deficiency.

We determined whether such putative novel biomarkers were also present in other samples of our cohort by systematically detecting all aberrant non‐biomarker metabolites present in an enriched biomarker pathway (Table 2). We report the remaining 142 metabolites by IMD as putative novel biomarkers pending analytical and functional validation and literature review.

**TABLE 2:** Metabolite set enrichment analysis (MSEA) pathway enrichment can aid in the discovery of novel IMD biomarker metabolites*.* For each MSEA‐enriched pathway containing a known biomarker, we identified all other metabolites in the pathway associated with an enriched feature following correction for multiple testing (Bonferroni–Holm *p* < 0.05, right column). We removed known biomarkers from this list (Table 1). We report the remainder as putatively novel biomarkers for the indicated IMD pending analytical and functional evaluation and literature review

Sample	Diagnosis	Enriched biomarker pathway	Putative novel metabolite biomarker
RadboudUMC_1	3‐Ureidopropionase deficiency	SMP0000007; SMP0000046; hsa00240; hsa00410	Carnosine (HMDB0000033/C00386); 1,3‐diaminopropane (HMDB0000002/C00986); carbon dioxide (HMDB0001967); flavin mononucleotide (HMDB0001520); l‐histidine (HMDB0000177/C00135); malonic semialdehyde (HMDB0011111/C00222); uracil (HMDB0000300/C00106); pantothenic acid (HMDB0000210/C00864); beta‐alanine (HMDB0000056/C00099); dUMP (HMDB0001409/C00365); phosphate (HMDB0001429); thymidine (HMDB0000273/C00214); cytidine monophosphate (HMDB0000095/C00055); dCDP (HMDB0001245/C00705); uridine (HMDB0000296/C00299); ureidosuccinic acid (HMDB0000828/C00438); deoxycytidine (HMDB0000014/C00881); l‐glutamine (HMDB0000641/C00064); phosphoric acid (HMDB0002142); 3‐aminoisobutanoic acid (HMDB0003911); 3’‐CMP (C05822); pseudouridine (C02067); uridine 2′,3′‐cyclic phosphate (C02355); (*R*)‐b‐aminoisobutyric acid (C01205); (*R*)‐5,6‐dihydrothymine (C21028); (*R*)‐3‐ureidoisobutyrate (C21029); gamma‐aminobutyric acid (C00334); 4‐aminobutyraldehyde (C00555); spermidine (C00315); apermine (C00750)
RadboudUMC_2	Cystathionine ß‐synthase deficiency	SMP0000033; hsa00270; hsa01210	*S*‐Adenosylhomocysteine (HMDB0000939/C00021); 5′‐methylthioadenosine (HMDB0001173/C00170); betaine (HMDB0000043); homocysteine (HMDB0000742/C00155); 1‐aminocyclopropanecarboxylic acid (C01234); 3‐sulfinoalanine (C00606); *O*‐acetyl‐l‐homoserine (C01077); (*S*)‐3‐methyl‐2‐oxopentanoic acid (C00671); l‐valine (C00183); pentahomomethionine (C17229); 3‐hydroxy‐3‐methyl‐2‐oxobutanoic acid (C04181); isopropylmaleate (C02631); ketoleucine (C00233); (E)‐4‐hydroxyphenylacetaldehyde oxime (C04350); citraconic acid (C02226); trihomomethionine (C17221); (*S*)‐2‐acetolactate (C06010); aminoadipic acid (C00956)
RadboudUMC_3	Histidinemia	hsa00340	Imidazole‐4‐acetaldehyde (C05130); imidazoleacetic acid riboside (C05131); 4‐imidazolone‐5‐propionic acid (C03680); gamma‐l‐glutamyl‐*S*‐(hercyn‐2‐yl)‐l‐cysteine *S*‐oxide (C20995); l‐glutamic acid (C00025)
RadboudUMC_4	Hyperlysinemia, Type I	hsa00300; **hsa00310**; hsa00780	(2*R*,3*R*)‐3‐Methylornithine (C20277); diaminopimelic acid (C00666); meso‐2,6‐diaminoheptanedioate (C00680); homoisocitrate (C05662); homocitric acid (C01251); *N*6‐acetyl‐l‐lysine (C02727); 2,5‐diaminohexanoate (C05161); (3*S*,5*S*)‐3,5‐diaminohexanoate (C01186); d‐lysine (C00739); d‐lysopine (C04020); (3*S*)‐3,6‐diaminohexanoate (C01142); pimelic acid (C02656)
RadboudUMC_31	Hyperlysinemia, Type I	hsa00310	*N*6‐acetyl‐l‐lysine (C02727); 2,5‐diaminohexanoate (C05161); (3*S*,5*S*)‐3,5‐diaminohexanoate (C01186); d‐lysine (C00739); d‐lysopine (C04020); (3*S*)‐3,6‐diaminohexanoate (C01142)
RadboudUMC_5	Hyperprolinemia, Type II	SMP0000020; **hsa00330**; hsa01230; hsa02010; hsa04974	d‐proline (HMDB0003411/C00763); 1‐pyrroline‐5‐carboxylic acid (HMDB0001301/C03912); 2‐oxoarginine (C03771); 4‐guanidinobutanamide (C03078); subaphylline (C10497); l‐phosphoarginine (C05945); *S*‐adenosylmethionine (C00019); (*S*)‐2‐acetolactate (C06010); *N*‐acetyl‐l‐glutamate 5‐semialdehyde (C01250); norfloxacin (C06687); sparfloxacin (C07662); phenol (C00146)
RadboudUMC_32	Hyperprolinemia, Type II	hsa00330	2‐Oxoarginine (C03771); (*S*)‐1‐pyrroline‐5‐carboxylate (C03912); subaphylline (C10497); d‐proline (C00763); *S*‐adenosylmethionine (C00019); 4‐acetamidobutanoic acid (C02946)
RadboudUMC_14	3β‐Hydroxy‐∆5‐C27‐steroid dehydrogenase deficiency	SMP0000035; hsa00120	Cholic acid (HMDB0000619/C00695); 7alpha‐hydroxy‐3‐oxo‐4‐cholestenoate (HMDB0012458/C17337)
RadboudUMC_33	Maple syrup urine disease	SMP0000032; hsa00280; hsa01210; hsa05230	l‐Valine (HMDB0000883/C00183); (*S*)‐3‐hydroxyisobutyric acid (HMDB0000023/C06001); (*S*/*R*)‐b‐aminoisobutyric acid (HMDB0002166/C03284/C01205); (*S*)‐3‐methyl‐2‐oxopentanoic acid (C00671); 3‐hydroxyisovalerate (C20827); l‐tryptophan (C00078); l‐phenylalanine (C00079); phenylpyruvic acid (C00166); (*R*)‐2,3‐dihydroxy‐isovalerate (C04272); 2‐oxo‐6‐methylthiohexanoic acid (C17216); glucocochlearin (C08407); citraconic acid (C02226); (*R*)‐2‐methylmalate (C02612); d‐erythro‐3‐methylmalate (C06032); l‐lysine (C00047); 2‐methylpropyl glucosinolate (C17256); l‐malic acid (C00149); d‐glucose (C00031); fumaric acid (C00122); l‐lactic acid (C00186)
RadboudUMC_34	Maple syrup urine disease	SMP0000032; hsa00280; hsa00290; hsa01230	(*S*)‐3‐Hydroxyisobutyric acid (HMDB0000023/C06001); (*S*)‐3‐methyl‐2‐oxopentanoic acid (C00671); 3‐hydroxyisovalerate (C20827); citraconic acid (C02226); (*R*)‐3‐hydroxy‐3‐methyl‐2‐oxopentanoate (C14463); (*S*)‐2‐aceto‐2‐hydroxybutanoic acid (C06006); diaminopimelic acid (C00666); meso‐2,6‐diaminoheptanedioate (C00680); *S*‐adenosylmethionine (C00019); sedoheptulose 7‐phosphate (C05382)
RadboudUMC_46	Phenylketonuria	hsa00360	3‐Hydroxyphenylacetic acid (C05593); vanillin (C00755); 3‐(3‐hydroxyphenyl)propanoic acid (C11457); 1‐phenyl‐1,2‐propanedione (C17268); ortho‐hydroxyphenylacetic acid (C05852); phenylacetylglycine (C05598); phenylacetic acid (C07086); *trans*‐cinnamic acid (C00423); *p*‐hydroxyphenylacetic acid (C00642); 3‐(2‐hydroxyphenyl)propanoic acid (C01198); d‐phenyllactic acid (C05607)
RadboudUMC_47	Phenylketonuria	hsa00360	1‐Phenyl‐1,2‐propanedione (C17268); phenylacetic acid (C07086); *trans*‐cinnamic acid (C00423)
RadboudUMC_49	Phenylketonuria	**hsa00360**; hsa04974; hsa00970	Phenylacetic acid (C07086); *trans*‐cinnamic acid (C00423); 3‐hydroxyphenylacetic acid (C05593); Vanillin (C00755); *p*‐hydroxyphenylacetic acid (C00642); 1‐phenyl‐1,2‐propanedione (C17268); ortho‐hydroxyphenylacetic acid (C05852); l‐arginine (C00062); l‐glutamine (C00064); l‐lysine (C00047); indole (C00463)
RadboudUMC_50a	Phenylketonuria	hsa00360	Phenylacetic acid (C07086); *trans*‐cinnamic acid (C00423); 2‐hydroxy‐6‐ketononatrienedioate (C12624); 1‐phenyl‐1,2‐propanedione (C17268)
RadboudUMC_53	UMP synthase deficiency	SMP0000046; hsa00240	Uracil (HMDB0000300/C00106); hydroxypropionic acid (C01013)
RadboudUMC_55	Xanthinuria, Type II	SMP0000050; hsa00230; hsa00232	SAICAR (HMDB0000797/C04823); (*R*/*S*)(−)‐allantoin (C02348/C02350); 5‐hydroxy‐2‐oxo‐4‐ureido‐2,5‐dihydro‐1*H*‐imidazole‐5‐carboxylate (C12248); 1,3,7‐trimethyluric acid (C16361)

**TABLE 3:** The biomarker pathways that were prioritised for each inherited metabolic disorder (IMD) in the data by our metabolite set enrichment analysis (MSEA) implementation. Bold biomarkers were not detected in all samples; Table S4A shows the exact samples in which the biomarker was or was not measured. Table S4B gives a more extensive list of all theoretically biomarker pathways available, including medication and disease pathways

IEM	OMIM	Pathway name	Aberrant biomarkers	Enriched
3‐Ureidopropionase deficiency	613161	Beta‐alanine metabolism	Ureidopropionic acid	1/1
		Pyrimidine metabolism	**Dihydrothymine**; ureidopropionic acid; ureidoisobutyric acid	1/1
		Pyrimidine metabolism	Ureidopropionic acid	1/1
		Beta‐alanine metabolism	Ureidopropionic acid	1/1
3β‐Hydroxy‐∆5‐C27‐steroid dehydrogenase deficiency	607765	Bile acid biosynthesis	3b,7a‐Dihydroxy‐5‐cholestenoic acid	1/1
		Primary bile acid biosynthesis	3b,7a‐Dihydroxy‐5‐cholestenoic acid	1/1
Cystathionine ß‐synthase deficiency	236200	Betaine metabolism	l‐Methionine	1/1
		Methionine Metabolism	l‐Methionine; methionine sulphoxide	1/1
		Cysteine and methionine metabolism	l‐Methionine; methionine sulphoxide	1/1
		2‐Oxocarboxylic acid metabolism	l‐Methionine	1/1
Histidinemia	235800	Histidine metabolism	Histidine; imidazole lactic acid	1/1
Hyperlysinemia, Type I	238700	Biotin metabolism	Lysine	1/2
		Lysine degradation	Lysine; **l‐pipecolic acid**	1/2
		tRNA charging: lysine	Lysine	2/2
		Lysine biosynthesis	Lysine	1/2
		Lysine degradation	Lysine; **l‐pipecolic acid**	2/2
		Biotin metabolism	Lysine	1/2
Hyperprolinemia, Type II	239510	Arginine and proline metabolism	Proline; 1‐pyrroline‐2‐carboxylic acid	1/2
		tRNA charging: proline	Proline	2/2
		Arginine and proline metabolism	Proline; 1‐pyrroline‐2‐carboxylic acid; pyrrole‐2‐carboxylic acid	2/2
		Biosynthesis of amino acids	Proline	1/2
		ABC transporters	Proline	1/2
		Protein digestion and absorption	Proline	1/2
		Mineral absorption	Proline	1/2
Maple syrup urine disease	248600	Valine, leucine and isoleucine degradation	Leucine; isoleucine; ketoleucine; *2‐ketoisovaleric acid*;3‐methyl‐2‐oxovaleric acid	2/2
		Valine, leucine and isoleucine degradation	Leucine; isoleucine; ketoleucine; *2‐ketoisovaleric acid*	2/2
		Valine, leucine and isoleucine biosynthesis	Leucine; isoleucine; ketoleucine; *2‐ketoisovaleric acid*	2/2
		2‐Oxocarboxylic acid metabolism	Leucine; isoleucine; ketoleucine; *2‐ketoisovaleric acid*	2/2
		Biosynthesis of amino acids	Leucine; isoleucine; ketoleucine; *2‐ketoisovaleric acid*	1/2
		Central carbon metabolism in cancer	Leucine; isoleucine	1/2
Methionine adenosyltransferase I/III deficiency	250850	Spermidine and spermine biosynthesis	l‐methionine	1/2
		tRNA charging: methionine	l‐methionine	2/2
Phenylketonuria	261600	Phenylalanine and tyrosine metabolism	Phenylalanine	2/9
		tRNA charging: phenylalanine	Phenylalanine	8/9
		Phenylalanine metabolism	Phenylalanine; *N‐acetyl‐l‐phenylalanine*	4/9
		Phenylalanine, tyrosine and tryptophan biosynthesis	Phenylalanine	1/9
		Aminoacyl‐tRNA biosynthesis	Phenylalanine	1/9
		Protein digestion and absorption	Phenylalanine	1/9
		Mineral absorption	Phenylalanine	1/9
UMP synthase deficiency	258900	Pyrimidine metabolism	Orotic acid; dihydroorotic acid	1/1
		Pyrimidine metabolism	Orotic acid; dihydroorotic acid	1/1
Xanthinuria, Type II	603592	Purine metabolism	Xanthosine; xanthine; *uric acid*	1/2
		Purine metabolism	Xanthosine; xanthine; *uric acid*; 5‐hydroxyisourate	1/2
		Caffeine metabolism	Xanthosine; xanthine	2/2
Glutaric aciduria Type I	231670	Fatty acid degradation	**Glutaric acid**	1/1

**FIGURE 3:**
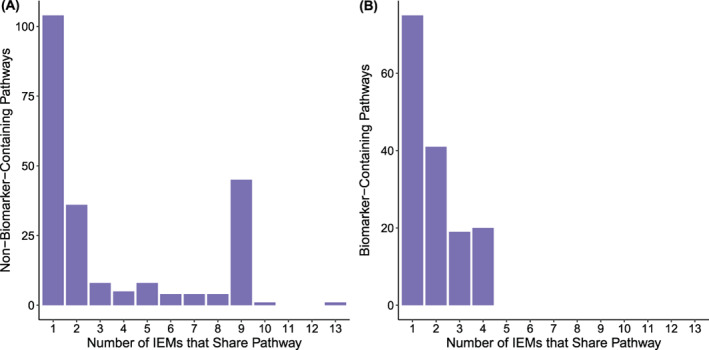
Biomarker‐containing pathways are relatively inherited metabolic disorder (IMD) specific. (A) The counts of biomarker‐containing and non‐biomarker‐containing pathways across 64 samples were plotted by the number of IMDs that share enrichment of each pathway. Biomarker‐containing pathways are significantly more IMD‐specific overall than non‐biomarker‐containing pathways (Wilcoxon *p* = 0.016). We indicate a set of pathways enriched by common non‐steroidal anti‐inflammatory drugs (NSAIDs) taken by a subset of our cohort with asterisk symbol (Table S5). If we remove these confounding pathways, biomarker‐containing pathways are not significantly more IMD specific (Wilcoxon *p =* 0.13)

Although we prioritised relevant IMD pathways with our method, we also found many (mean, 20; SD, 22) other pathways enriched. To better understand these pathways, we explored the distribution of all enriched pathways across the 29 IMDs in our dataset. In addition, we showed that biomarker pathways are significantly more IMD‐specific than non‐biomarker pathways, and were shared across a maximum of 4 IMDs versus 13 for non‐biomarker pathways (Figure 3, Wilcoxon *p =* 0.016). By binning enriched pathways based on the number of IMDs that share them, we were also able to detect 40 enriched pathways shared across 9 IMDs (Figure 3A, indicated by *). We determined that these pathways were shared not just across the same IMDs, but also the same set of patients within our dataset, and were able to determine that 37 of the 40 pathways are associated with non‐steroidal anti‐inflammatory drugs (Table S5). We anticipate that other common, non‐IMD‐specific dietary and medication effects may also be detectable in this way.

## 4 DISCUSSION

MSEA prioritises biologically relevant metabolites from high‐dimensionality NGMS data by leveraging pathway context. We show that MSEA consistently prioritises known IMD biomarkers by pathway or cluster rank when biomarkers are associated with pathways, and that it is complementary to a feature‐based filtering approach. We also demonstrate the two specific advantages of MSEA over traditional analysis by feature‐based prioritisation or an IMD panel: MSEA does not use known biomarker information as a prior for pathway enrichment, and MSEA reports not just biomarker metabolites but also their enriched pathway context. Using the first, we can describe characteristics of MSEA‐enriched biomarker pathways that allow data reduction without a genetic or metabolomic prior; using the second, we can report putative novel biomarker metabolites in IMDs with a previously characterised biomarker to improve diagnosis and clinical follow‐up of patients with these disorders. One of these putative novel biomarkers for CBS deficiency, 5′‐methylthioadenosine, is currently validated in CBS‐deficiency patients using orthogonal methods and was previously reported to be found in a mouse model of CBS–homocystinuria.^18^

The main impediment to the routine use of UM in IMD diagnostics is the vast amount of data generated per run. Manual processing and analysis of this data is often not feasible, and the considerable amount of biological and technical noise in mass spectrometry data makes prioritisation of clinically relevant metabolites challenging. MSEA addresses this problem by using feature counts to prioritise a small number of biologically‐relevant pathways and their associated metabolites. MSEA does not use metabolite biomarker status in enrichment, and this independence allows analysis of patients with unresolved or novel IMDs, and the identification of novel biomarkers for previously characterised IMDs. These characteristics make MSEA unique in NGMS data analysis and allow its broad application on a variety of data sets.

However, MSEA is very dependent on the fidelity and completeness of pathway annotations in major databases like SMPDB and KEGG.^9^ For 6 out of 29 IMDs, known biomarker pathways could not be enriched as they were missing from the pathway databases. In some cases we could find an IMD specific pathway, but IMD biomarker metabolites were still missing from those pathways. For example, in the 3‐hydroxy‐3‐methylglutaryl‐CoA lyase deficiency pathway (SMP0000138) our biomarkers are not yet included, but we can verify that they ought to be based on the corresponding (co)enzymes that are present (Table S6). In addition, interpreting MSEA‐enriched pathways with no known IMD biomarkers in a clinical context is still a major challenge despite some data reduction approaches as described here. Medication and diet are major confounders and will also enrich pathways in MSEA. Distinguishing between these pathways and those of diagnostic significance is predicated upon thorough patient phenotyping and completeness of online databases and drug reference panels, which are also often incomplete.

There are several ways in which MSEA could be extended to improve feature prioritisation or IMD coverage. First, if the IMD is not properly described through pathway information as discussed previously, metabolite sets could be made based on other functional assays or biological data. Second, data pre‐processing could be improved by normalising feature intensities, incorporating feature quality filters, and reducing incorrect annotations by using an adduct annotation algorithm like CAMERA.^19^ This pre‐processing could potentially further reduce the size of MSEA output while retaining biologically important metabolites. Normalisation could also potentially allow the identification of additional features that could enrich biomarker pathways currently not enriched. Third, the incorporation of covariates relating to patient gender, age, and treatment status when known could be beneficial.

In the future, we anticipate that MSEA can be a useful addition to NGMS data analysis in a diagnostic setting. Furthermore, through its ability to identify novel biomarkers, MSEA will allow for expansion of existing diagnostic IMD panels and increase the diagnostic yield of NGMS generally. We show this by example in this paper in CBS deficiency, but we think this is one of the major advantages of a pathway‐based enrichment approach and more novel biomarkers will be functionally validated from MSEA output on this dataset and others. More broadly, we see advantages in pathway‐based approaches both for filtering metabolite sets that are not disease‐associated and for identifying relevant disease‐associated metabolites for patient diagnostics.

In conclusion, we have created a MSEA method that prioritises metabolites in NGMS data by feature count‐based enrichment in biological pathways. Our method successfully reduces complexity of NGMS data while retaining diagnostic biomarkers for known IMDs. Furthermore, our method allows the identification of novel biomarkers for IMDs and offers the potential for diagnostic utility in patients suspected of an IMD. More broadly, MSEA exemplifies the utility of leveraging biological information in NGMS data reduction and can be applied in both diagnostics and research to easily identify biological relevant signals.

## AUTHOR CONTRIBUTIONS

*Conception and design*: Han G. Brunner, Karlien L. M. Coene, Christian Gilissen, Ron A. Wevers. *Method development*: Brechtje Hoegen. *Preprocessing of data*: Brechtje Hoegen and Purva Kulkarni. *Analysis and interpretation*: Udo F. H. Engelke, Juliet E. Hampstead and Brechtje Hoegen. *Writing and revising draft*: Juliet E. Hampstead and Brechtje Hoegen. *Reviewing draft*: Han G. Brunner, Karlien L. M. Coene, Christian Gilissen, Purva Kulkarni, Ron A. Wevers.

## CONFLICT OF INTEREST

The authors declare no conflict of interest.

## ETHICS STATEMENT

All patients and control subjects (or their guardians) registered their informed consent for the possible use of their leftover body fluid samples from clinical diagnostics for laboratory method validation purposes in their electronic patient record, in agreement with institutional and national legislation.

## Supporting information


**FIGURE S1** ROC curve, intensity outperforms absolute fold change and Bonferroni–Holm feature *p*‐value for the ranking of known biomarkers
**FIGURE S2**. Boxplot of aberrant features versus annotated features versus pathway features
**FIGURE S3**. Boxplot biomarkers have a lower row index in MSEA output than in raw feature output sorted by intensity
**FIGURE S4**. Boxplot clustering of enriched pathways promotes easy interpretation and reduces pathways enriched by the same aberrant features.
**FIGURE S5**. Histogram of pathway and clustered pathway biomarker ranks

**TABLE S1** Samples

**TABLE S2** Method parameters

**TABLE S3** Biomarkers

**TABLE S4** Cystathionine ß‐synthase deficiency (CBSD)

**TABLE S5** Pathways enriched for nine different IEMs, mostly nonsteroidal anti‐inflammatory drugs (NSAIDs) table

**TABLE S6** 3‐Hydroxy‐3‐methylglutaryl‐CoA lyase deficiency (HMCLD) pathway co‐enzymes and metabolites

## Data Availability

The data presented in this study are available upon reasonable request from the corresponding author. Data are not publicly available due to the terms of the ethical approval.
