# Supplementary material for: Application of metabolite set enrichment analysis on untargeted metabolomics data prioritises relevant pathways and detects novel biomarkers for inherited metabolic disorders
Source: J Inherit Metab Dis. 2022 May 22;45(4):682–95. doi: 10.1002/jimd.12522 (PMC9544878; doi:10.1002/jimd.12522)
Supplement: Supplementary file 1 — FIGURE S1 ROC curve, intensity outperforms absolute fold change and Bonferroni–Holm feature p‐value for the ranking of known biomarkers FIGURE S2. Boxplot of aberrant features versus annotated features versus pathway features FIGURE S3. Boxplot biomarkers have a lower row index in MSEA output than in raw feature output sorted by intensity FIGURE S4. Boxplot clustering of enriched pathways promotes easy interpretation and reduces pathways enriched by the same aberrant features. FIGURE S5. Histogram of pathway and clustered pathway biomarker ranks [file JIMD-45-682-s004.pdf]

Supplementary Overview

- **Supplementary Table 1: Samples**
    - **A:** Details on data exclusion between Coene *et. al.* and this study
    - **B:** Extensive/in depth overview of included patients in this study including batch/instrument data
- **Supplementary Table 2:** Method parameters
    - **A**: MSConvert
    - **B**: XCMS
- **Supplementary Table 3: Biomarkers**
    - **A**: List of available IEM Pathways
    - **B**: List of IEM Biomarkers used in this study
    - **C**: List of Pathways containing IEM Biomarkers
    - **D**: List with biomarker ranks per sample.
- **Supplementary Table 4:** Cystathionine ß-synthase deficiency (CBSD)
    - **A:** Pathway table containing MSEA results.
    - **B:** Cluster table containing clustered MSEA results.
    - **C:** Feature table containing hsa00270 features
    - **D:** 1-Aminocyclopropanecarboxylic acid, explanation why annotation is false positive.
    - **E:** O-Acetyl-L-homoserine, explanation why annotation is false positive.
    - **F:** 3-Sulfinoalanine, explanation why annotation is false positive.
- **Supplementary Table 5**: Pathways enriched for 9 different IEMs, mostly Nonsteroidal anti-inflammatory drugs (NSAIDs) table
- **Supplementary Table 6**: 3-Hydroxy-3-methylglutaryl-CoA Lyase Deficiency (HMCLD) pathway co-enzymes and metabolites
- **Supplementary Figure 1:** ROC curve, intensity outperforms absolute fold change and Bonferroni-Holm feature p-value for the ranking of known biomarkers
- **Supplementary Figure 2:** Boxplot of aberrant features vs annotated features vs pathway features.
- **Supplementary Figure 3**: Boxplot Biomarkers have a lower row index in MSEA output than in raw feature output sorted by intensity
- **Supplementary figure 4:** Boxplot Clustering of enriched pathways promotes easy interpretation and reduces pathways enriched by the same aberrant features.
- **Supplementary figure 5:** Histogram of pathway and clustered pathway biomarker ranks.

Supplementary Figures

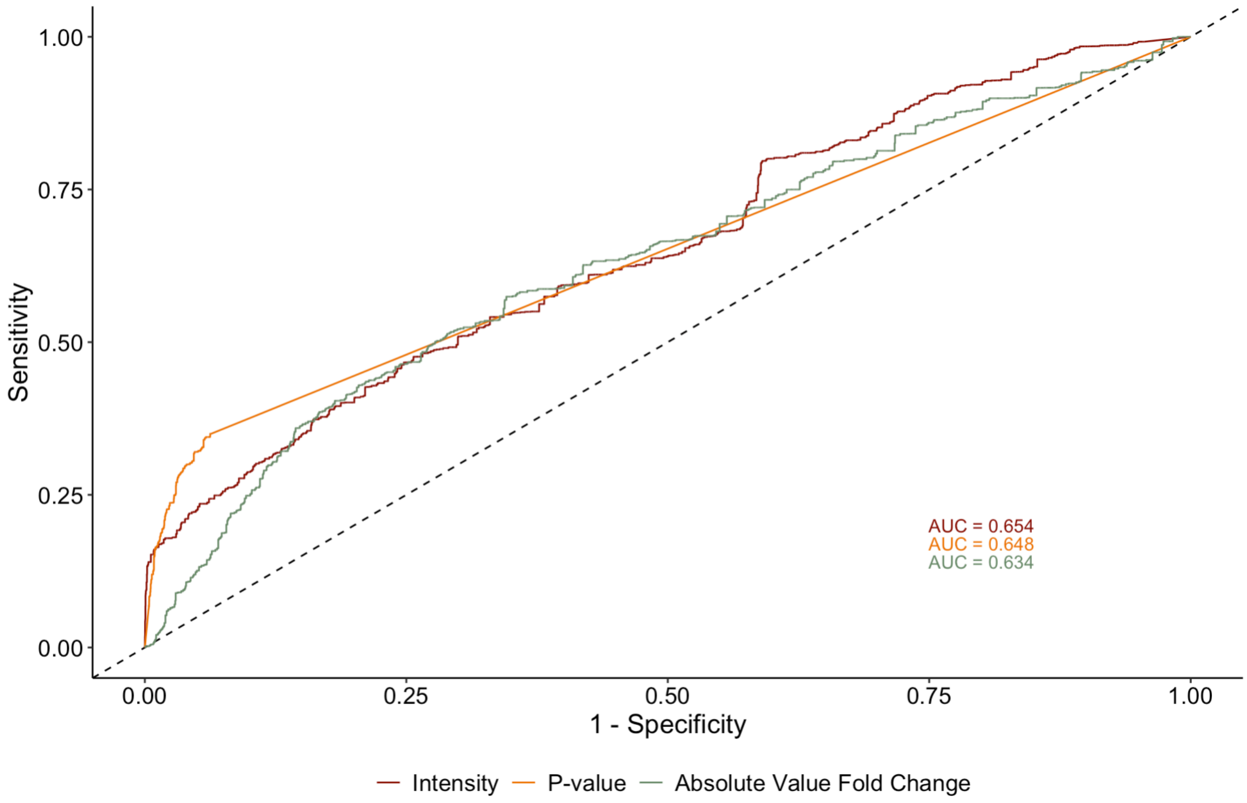

Supplementary Figure 1. ***Intensity outperforms absolute value fold change and Bonferoni-Holm corrected p-value for ranking biomarker-associated features.*** Biomarker-associated features were ranked by intensity (red), p-value (orange), and absolute value fold change (light green). The sensitivity and specificity of biomarker detection was calculated at different intensity levels, p-value cutoffs, and fold change cutoffs. AUCs are summarised below the plot in text. Although all three methods are comparable, in our dataset feature intensity best prioritises biomarker-associated features.

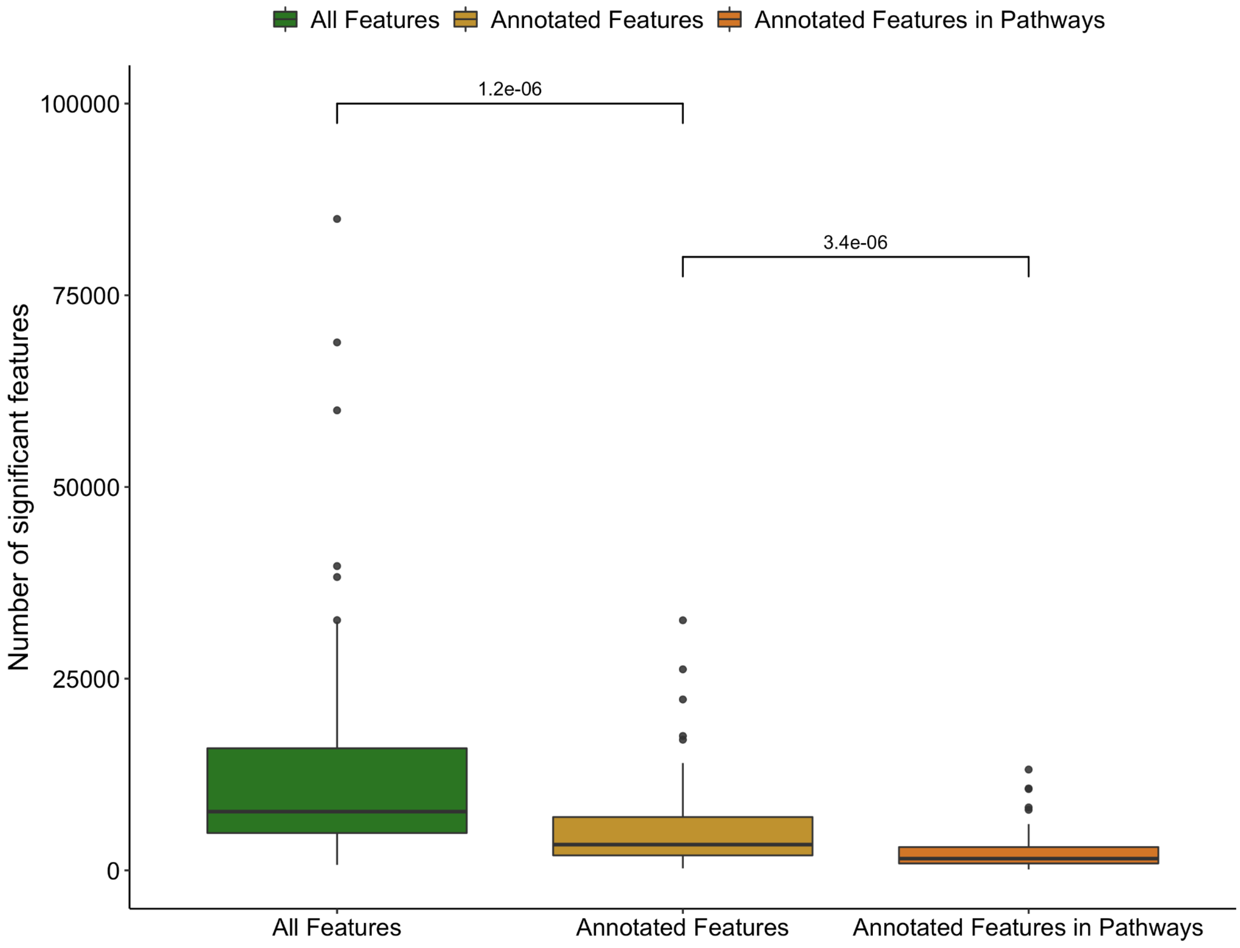

Supplementary Figure 2. ***Pathway-associated features are a subset of the total detected features per sample.*** The boxplots above represent the total number of significant features (Benjamini-Hochberg $p < 0.05$, green), the number of significant features with a putative metabolite annotation in HMDB or KEGG (pink), and the number of significant features with a putative metabolite annotation associated with a pathway in SMPDB or KEGG (blue).

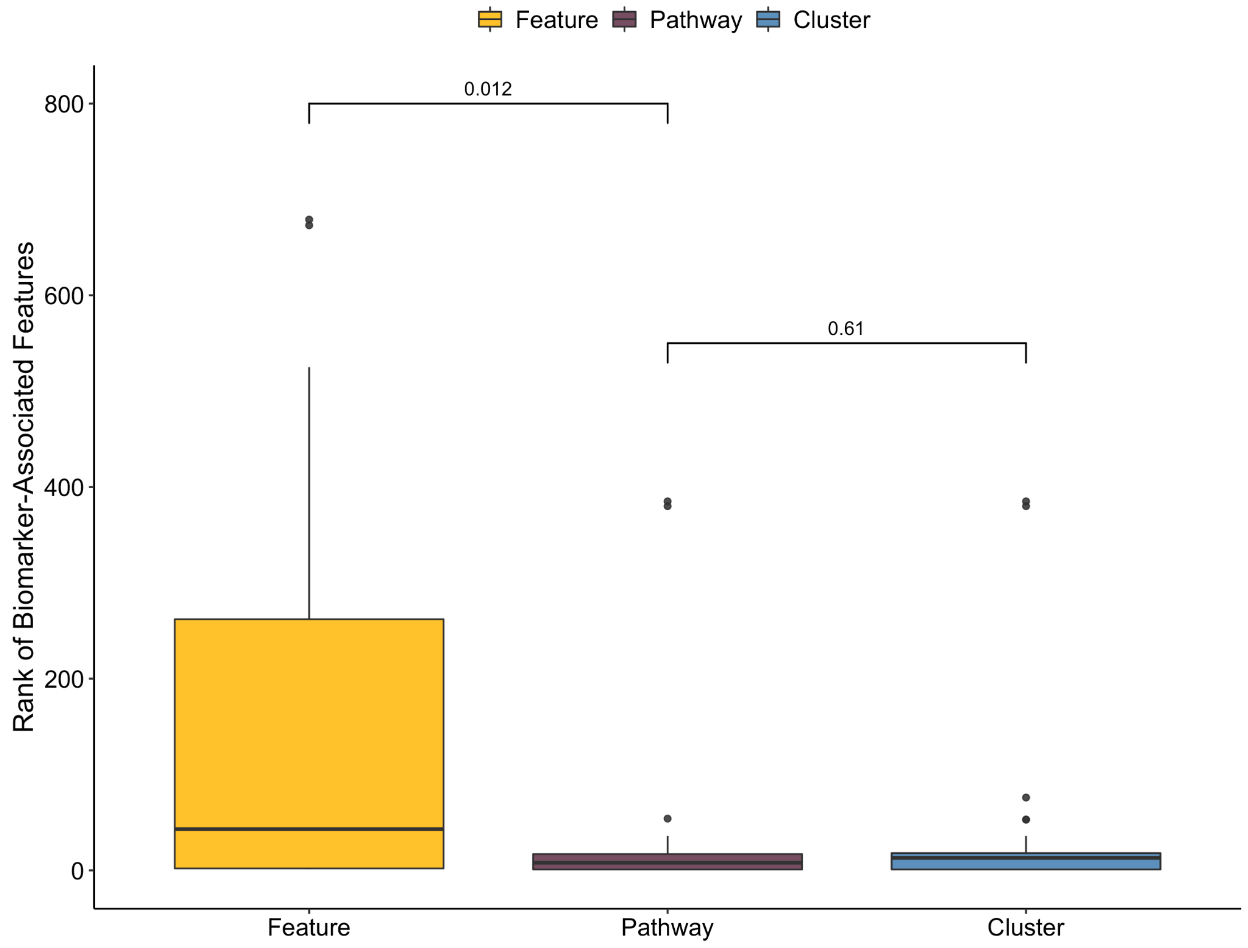

Supplementary Figure 3. ***MSEA output improves prioritisation of features with a putative biomarker metabolite annotation.*** The biomarker indexes of all biomarker-associated features present in an enriched pathway was computed per patient sample by ranking on feature intensity (Intensity), pathway p-value + feature intensity (Pathway), and cluster p-value + feature intensity (Cluster) (see **Methods**). The distribution of biomarker indexes between features and pathways is significantly different (Intensity, Pathway Wilcoxon p = 0.032). Clustering did not significantly improve feature biomarker rank (Wilcoxon p = 0.63).

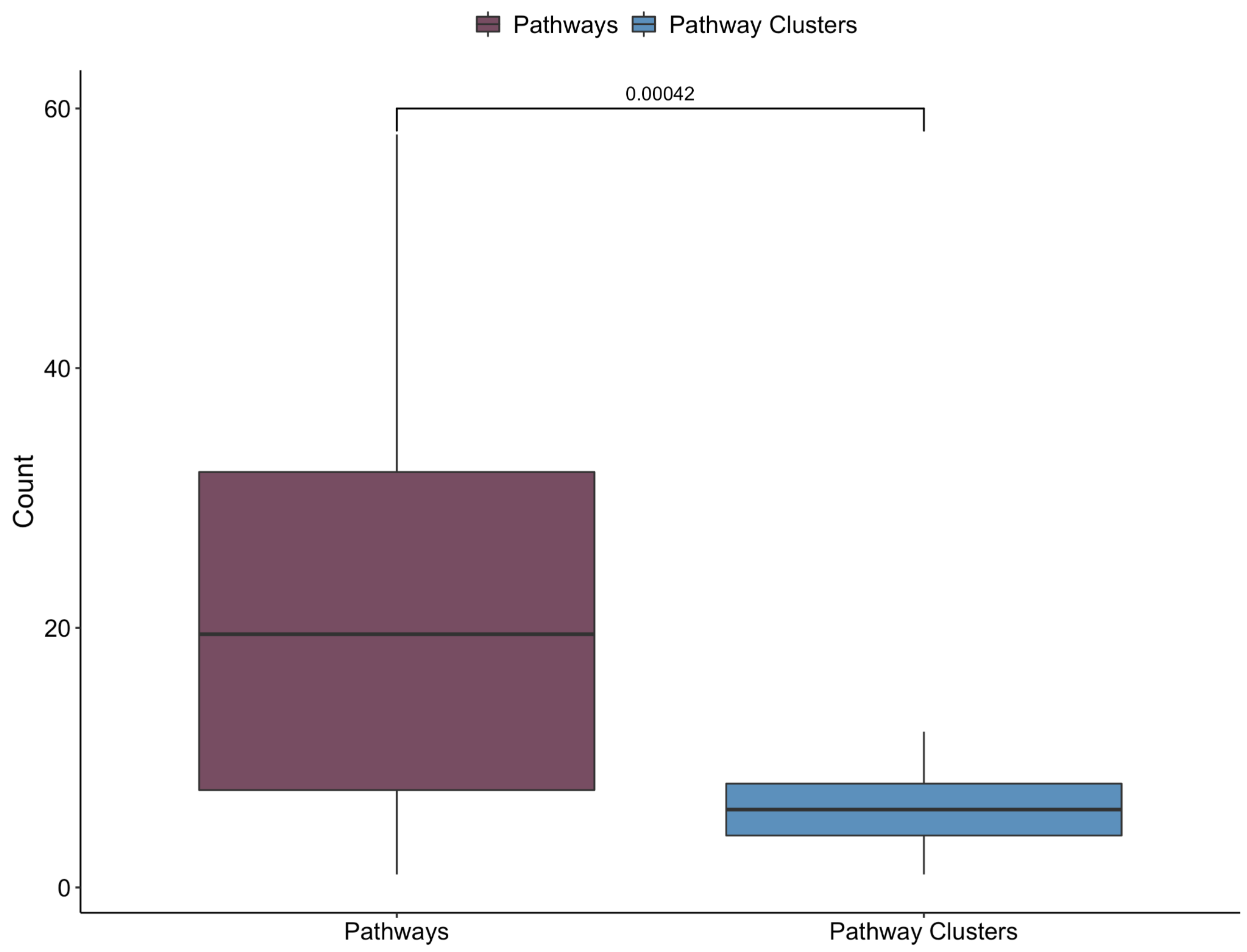

Supplementary Figure 4. ***Clustering of enriched pathways promotes easy interpretation and reduces pathways enriched by the same aberrant features.*** MSEA enriched pathways were clustered into pathway clusters as described in **Methods**. These pathway clusters eliminate redundancy in pathway information while still prioritising pathway-associated metabolite biomarkers (**Supplementary Figure 3**).

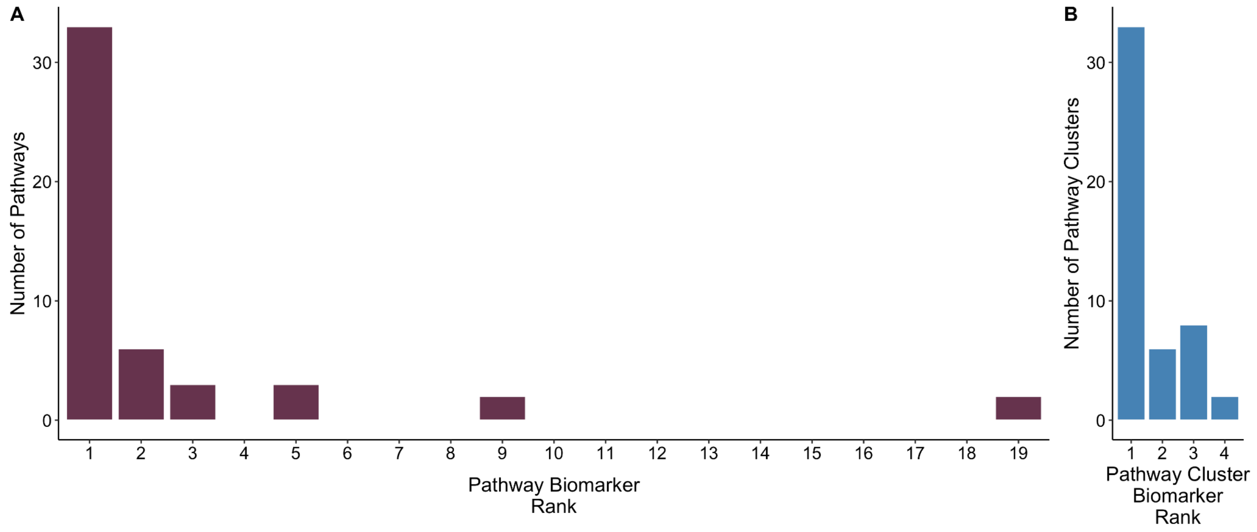

Supplementary Figure 5. ***Pathway and pathway cluster ranks of pathway-associated biomarkers from MSEA.*** Pathway and pathway cluster ranks were computed as described in **Methods**. The distribution of ranks of pathway-associated biomarkers is shown in purple. These ranks in clustered pathways are shown in blue.
